# Pancreatico-pleural fistula in chronic pancreatitis: A case report

**DOI:** 10.1097/MD.0000000000047864

**Published:** 2026-03-27

**Authors:** Wangli Qiu, Lianhai Li, Jia Fang, Jisheng Wang, Tianwen Lai

**Affiliations:** a Department of Respiratory and Critical Care Medicine, The First Dongguan Affiliated Hospital, Guangdong Medical University, Dongguan, China; b Department of General Surgery, The First Dongguan Affiliated Hospital, Guangdong Medical University, Dongguan, China.

**Keywords:** chronic pancreatitis, pancreatico-pleural fistula, pleural effusion

## Abstract

**Rationale:**

Pancreatico-pleural fistula (PPF) is a rare and easily misdiagnosed complication of chronic pancreatitis, often presenting with respiratory symptoms and leading to significant diagnostic delays.

**Patient concerns:**

A 49-year-old male with chronic pancreatitis and alcohol abuse presented with a 6-month history of recurrent cough, sputum, and chest tightness, initially misdiagnosed as inflammatory pleural effusion.

**Diagnoses:**

PPF was confirmed by left pleural effusion with high amylase (9372 U/L), MRI/magnetic resonance cholangiopancreatography revealing a pancreatic pseudocyst and a fistula tract from the pancreatic tail to the left pleural cavity.

**Interventions:**

The patient underwent laparoscopic excision of the fistula tract, suture closure of the pancreatic end, and external drainage.

**Outcomes:**

Postoperative drainage fluid amylase remained elevated (183,940 U/L), indicating persistent leakage. The patient was discharged with an abdominal drain. At 2-month follow-up, no effusion recurrence was observed.

**Lessons:**

PPF should be considered in patients with chronic pancreatitis and unexplained pleural effusion. Magnetic resonance cholangiopancreatography is effective for noninvasive diagnosis. Early surgical intervention is recommended if short-term medical management fails to prevent complications and reduce morbidity.

## 1. Introduction

Pancreatico-pleural fistula (PPF) is a rare complication that usually occurs as a secondary condition to chronic pancreatitis or pancreatic injury in alcoholic patients. The incidence of PPF occurs in 0.4% of patients with chronic pancreatitis.^[1]^ Pathogenesis typically involves pancreatic duct leakage and incomplete or ruptured pseudocyst formation, causing pancreatic enzyme leakage, erosion of the retroperitoneum, leading to the formation of fluid in the posterior peritoneum.^[2,3]^ This process leads to fluid accumulation in the posterior peritoneum, which can subsequently traverse the esophageal hiatus or aortic hiatus into the mediastinum, establishing communication with the pleural cavity and culminating in PPF formation.^[4]^ This fistula track thus provides an amylase-rich secretion to the pleural cavity. Although elevated amylase levels in pleural effusions are usually associated with chronic pancreatitis, this phenomenon can also be caused by acute pancreatitis, esophageal perforation, or pancreatic malignancy.^[5–7]^ However, only PPF results in amylase levels in the pleural effusion exceeding 50,000 IU/L.^[8]^ The presence of pleural effusion caused by PPF often manifests as respiratory distress, cough, chest pain, and other chest symptoms and signs,^[9,10]^ which commonly result in missed diagnoses and diagnostic delays.^[2]^ We present a case of PPF with a diagnostic delay of up to 6 months.

## 2. Case presentation

### 2.1. Case history/examinations

A 49-year-old male with a long history of alcohol abuse was hospitalized due to recurrent cough, sputum production, and chest tightness for over 6 months. Six months ago, he was hospitalized in another hospital and diagnosed with inflammatory exudative pleural effusion after undergoing left thoracentesis and pleural biopsy, and was discharged after receiving antimicrobial, antitussive, and expectorant therapy. Despite treatment, the patient continued to experience recurrent cough and chest tightness after discharge. He has a history of pulmonary tuberculosis diagnosed over 10 years ago, which was managed with irregular, self-administered antituberculosis medication rather than a standard regimen, and a history of recurrent chronic pancreatitis for over 5 years, with 2 documented acute exacerbations. His vital signs were normal, and physical examination, routine blood tests, and tumor screening revealed no specific abnormalities.

**Figure 1.**
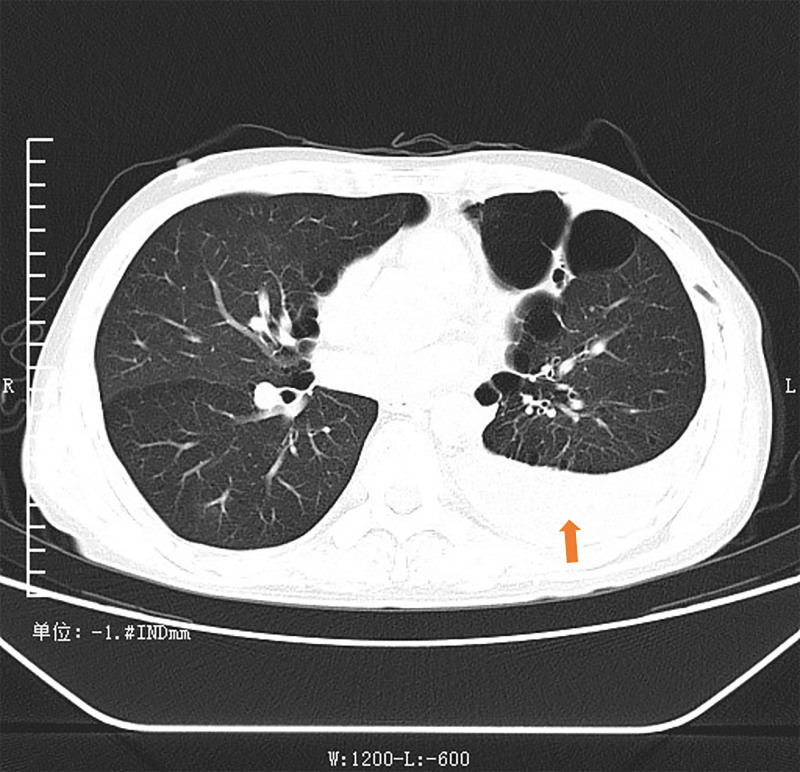
Axial lung window, with arrow indicating left pleural effusion.

**Figure 2.**
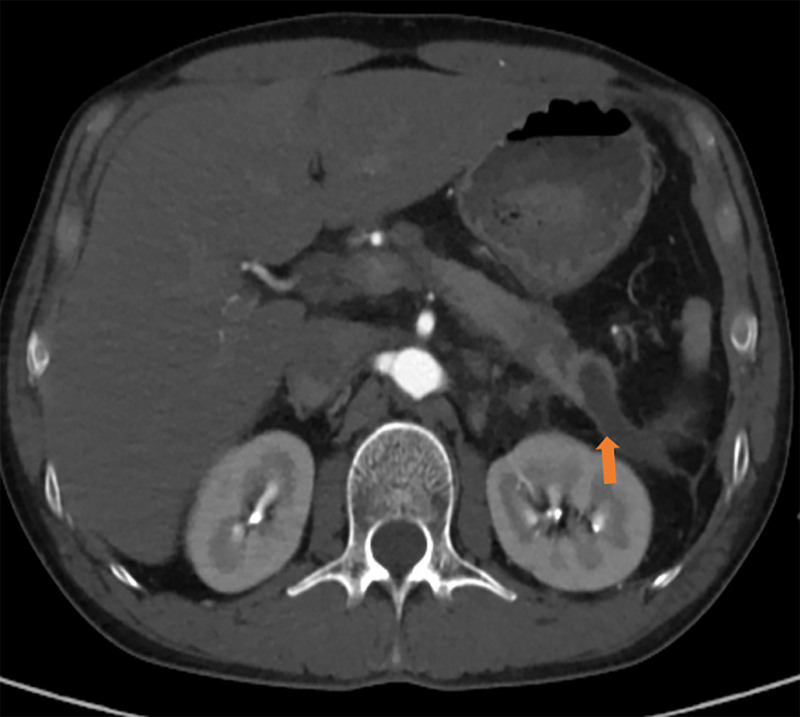
Contrast-enhanced MRI of the upper abdomen, the arrow indicates cystic lesion at the tail of the pancreas.

**Figure 3.**
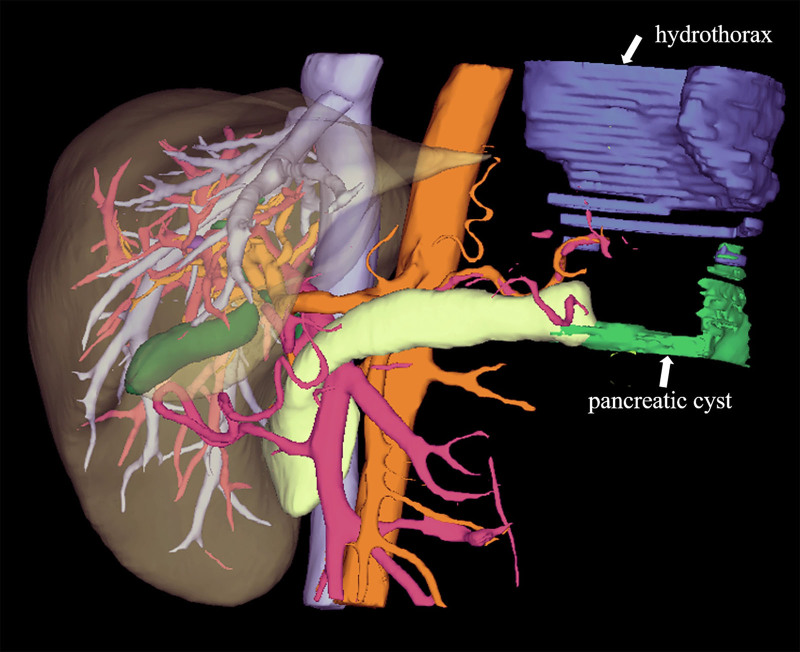
MRI three-dimensional reconstruction showing pancreatic cyst and pleural effusion caused by pancreatico-pleural fistula.

Ultrasound indicated a deep pleural effusion measuring approximately 68 mm deep at the 10th intercostal space on the left axillary line. A loculated pleural effusion on the left side was visible on chest CT (Fig. 1). Thoracentesis was performed for him, and dark red bloody pleural fluid was drained. The red blood cell count in the pleural fluid was 490.0 × 10^9^/L, amylase was elevated at 9372.00 U/L, and the nucleated cell count was 1726.0 × 10^6^/L. Further investigations to exclude common causes of exudative effusion were undertaken: the pleural fluid was positive for mucin, with elevated total protein (38.60 g/L). However, tests for tuberculosis were negative, including a qualitative TB-DNA assay and acid-fast bacillus smear. The lactate dehydrogenase level was 38.00 U/L. Pleural fluid cytology analysis revealed only a small number of eosinophils and mesothelial cells in a bloody background, with no evidence of malignant tumors. And the pathological examination of pleural biopsy under thoracoscopy showed predominantly fibrofatty tissue with chronic inflammation, granulation tissue formation, and eosinophil infiltration. Areas of fat necrosis, cellulose-like exudate, and proliferative mesothelial cell clusters were also observed. Upper abdominal MRI with contrast enhancement demonstrates an intrapancreatic cyst, multiple pseudocysts around the pancreas, exudation at the tail of the pancreas, and thickened adhesions to the left pleura (Fig. 2). Magnetic resonance cholangiopancreatography (MRCP) indicates dilation of the pancreatic duct. Three-dimensional reconstruction on MRI displays a fistulous tract extending from the cyst at the tail of the pancreas to the left cardiophrenic angle, connecting to the pleural cavity (Fig. 3).

### 2.2. Treatment

**Figure 4.**
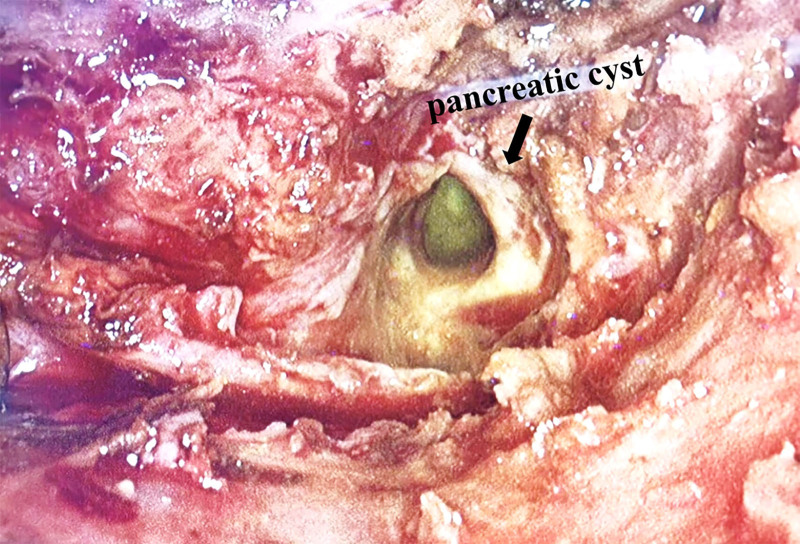
Intraoperative view of pancreatic cyst with yellow-green contents.

Laparoscopic exploration confirms the presence of a cystic ribbon-shaped mass measuring approximately 2.0 cm × 4.0 cm, containing yellow, crumbly necrotic material. It was located at the lower edge of the pancreatic tail and extends to the posterior aspect of the lower pole of the spleen, forming a sinus tract that ascends to the chest wall (Fig. 4). The pancreatic fistula tract was excised to separate it from the intra-abdominal fistulous system, disconnecting it from the mediastinum and pleural cavity, and the pancreatic end of the fistula was sutured. And the external drainage was performed from the pancreatic section.

### 2.3. Treatment procedure and follow up

**Figure 5.**
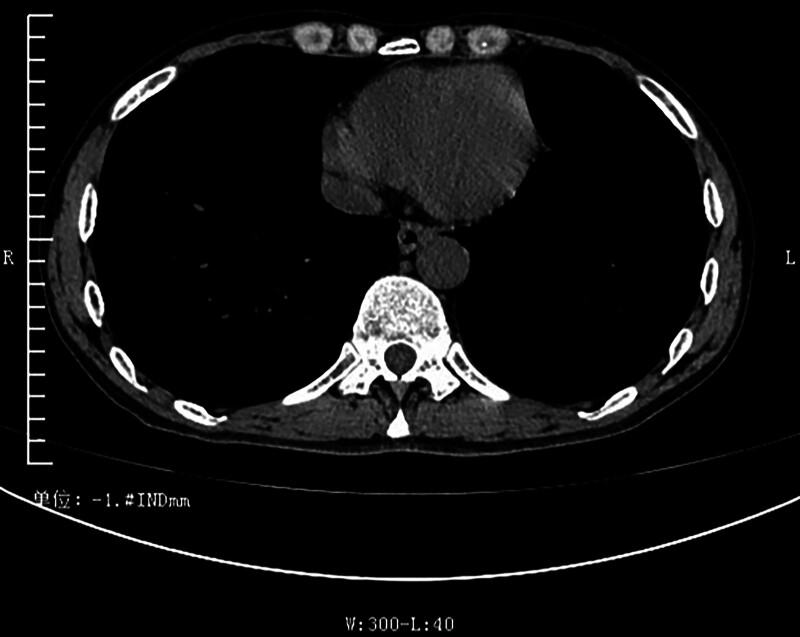
Axial mediastinal window. Follow-up chest CT at 2 months postoperatively showing no recurrence of pleural effusion.

The patient was ultimately diagnosed with PPF, and the postoperative drainage was timely, with good recovery. After surgical treatment, the patient showed good recovery. The drain from the pancreatic resection site was producing approximately 100 mL of fluid per day. The amylase level in the drained fluid was 183,940.00 U/L upon follow-up, indicating incomplete healing of the pancreatic resection site. Therefore, it was decided to continue with the drainage and discharge the patient with an abdominal drain in place. At the 2-month follow-up, no evidence of pleural or abdominal effusion recurrence was observed (Fig. 5).

## 3. Discussion

PPF is a rare but serious complication of chronic pancreatitis, and its diagnosis can be complex. This complexity arises because PPF symptoms are often nonspecific, with pulmonary symptoms frequently being more prominent than abdominal symptoms. As seen in our patient, this can lead to pulmonary symptoms being the primary complaint and may cause confusion with other respiratory conditions. Most pleural effusions associated with PPF are located in the left chest cavity and tend to be recurrent.^[11]^ Due to the high content of digestive enzymes in pancreatic fluid, an elevated amylase level in pleural effusion often suggests pancreatic-related disease. According to Tauseef et al, the average amylase level in pleural effusion is 47,362 U/L, with a range from 400 to 446,600 U/L.^[10]^ In this case, although the amylase level in the pleural fluid did not reach the previously mentioned threshold of 50,000 IU/L, this may be attributed to the reabsorption of amylase by the pleural cavity. Therefore, an unequivocal diagnosis of PPF should not be based solely on amylase levels. High amylase levels in pleural fluid may need to be differentiated from conditions such as acute pancreatitis, lung cancer, metastatic cancer, pneumonia, and esophageal perforation. CT scans, MRCP, and endoscopic retrograde cholangiopancreatography (ERCP) are crucial for diagnosing PPF, as they can identify associated abnormalities such as pancreatic pseudocysts and sometimes reveal the presence of a fistula.^[12]^ We recommend avoiding ERCP as the primary tool for confirming PPF due to its higher risk of infection. In contrast, MRCP is a noninvasive procedure that not only effectively reduces the risk of infection but also offers comparable accuracy in detecting pancreatic duct abnormalities, while providing clear visualization and delineation of the fistula and pancreatic duct anatomy.^[13]^

Due to the rarity of PPF, its management remains controversial. Medical management of PPF aims to maintain the pancreas in a state of low secretion to promote fistula closure. This includes nutritional support via parenteral nutrition or specialized diets to rest the pancreas, pharmacological inhibition of pancreatic secretion using somatostatin analogues, and adequate drainage of symptomatic pleural effusions. However, since it fails to address the underlying anatomical pathology of PPF, the efficacy of pharmacological therapy alone is highly limited.^[14]^

Current scientific evidence is primarily derived from case reports and case series, lacking randomized controlled trials for more systematic validation. Medication treatment has a failure rate of 59% to 69%,^[9,14]^ while surgical intervention shows a higher success rate compared to conservative treatment (94% vs 31%).^[2]^ Additionally, prolonged conservative medical treatment typically delays fistula healing and increases the risk of complications. Lipsett et al found that 80% of deaths due to nonsurgical treatment of PPF occurred in patients who had undergone conservative treatment for >3 weeks.^[15]^ ERCP is not only diagnostic but also has therapeutic benefits. The placement of a stent during ERCP can mechanically block the communication between the pancreatic duct and the fistula.^[12,16]^ Although ERCP may be successful in initial treatment, the progression of the disease often leads to subsequent complications that usually require surgical intervention.^[14]^ Currently, surgical intervention is typically considered a last resort, only pursued after medical and endoscopic treatments have failed. However, for cases with poor healing prospects where surgical intervention is not performed, delaying surgical intervention may lead to increased morbidity and mortality. Early surgical treatment of PPF may help in appropriately selected patients to save both cost and time, and prevent life-threatening complications resulting from ERCP failure, repeated thoracentesis, or prolonged chest drainage.

In conclusion, in patients with a history of chronic pancreatitis and long-term alcohol consumption, the emergence of pleural effusion accompanied by a marked increase in the amylase levels should alert to the potential presence of PPF, thereby avoiding diagnostic delays and unnecessary investigations. And we suggest that once PPF is diagnosed, surgery should be performed as soon as possible if short-term medication is ineffective in order to increase the success rate of treatment and minimize complications.

## Author contributions

**Conceptualization:** Wangli Qiu, Lianhai Li, Jia Fang, Jisheng Wang, Tianwen Lai.

**Data curation:** Wangli Qiu, Lianhai Li, Jia Fang, Jisheng Wang, Tianwen Lai.

**Formal analysis:** Wangli Qiu, Lianhai Li, Jia Fang, Jisheng Wang, Tianwen Lai.

**Funding acquisition:** Wangli Qiu, Lianhai Li, Jia Fang, Jisheng Wang, Tianwen Lai.

**Investigation:** Wangli Qiu, Lianhai Li, Jia Fang, Jisheng Wang, Tianwen Lai.

**Methodology:** Wangli Qiu, Lianhai Li, Jia Fang, Jisheng Wang, Tianwen Lai.

**Project administration:** Wangli Qiu, Lianhai Li, Jia Fang, Jisheng Wang, Tianwen Lai.

**Resources:** Wangli Qiu, Lianhai Li, Jia Fang, Jisheng Wang, Tianwen Lai.

**Software:** Wangli Qiu, Lianhai Li, Jia Fang, Jisheng Wang, Tianwen Lai.

**Supervision:** Wangli Qiu, Lianhai Li, Jia Fang, Jisheng Wang, Tianwen Lai.

**Validation:** Wangli Qiu, Lianhai Li, Jia Fang, Jisheng Wang, Tianwen Lai.

**Visualization:** Wangli Qiu, Lianhai Li, Jia Fang, Jisheng Wang, Tianwen Lai.

**Writing – original draft:** Wangli Qiu, Lianhai Li, Jia Fang, Jisheng Wang, Tianwen Lai.

**Writing – review & editing:** Wangli Qiu, Lianhai Li, Jia Fang, Jisheng Wang, Tianwen Lai.
